# Radiation-Induced Lymphopenia and Its Impact on Survival in Patients with Brain Metastasis

**DOI:** 10.3390/curroncol31080340

**Published:** 2024-08-09

**Authors:** Naoko Ishida, Yukinori Matsuo, Junki Fukuda, Aritoshi Ri, Saori Tatsuno, Takuya Uehara, Masahiro Inada, Tomohiro Matsuura, Hiroshi Doi, Kiyoshi Nakamatsu, Makoto Hosono

**Affiliations:** 1 Department of Radiation Oncology, Faculty of Medicine, Kindai University, 377-2 Onohigashi, Osakasayama 589-8511, Osaka, Japan; 2 Department of Radiation Oncology, Yamato Takada Municipal Hospital, 1-1 Isonokita-cho, Yamatotakada 635-0094, Nara, Japan; 3 Department of Radiation Oncology, Fuchu Hospital, 1-10-17 Hiko-cho, Izumi 594-0076, Osaka, Japan

**Keywords:** lymphopenia, brain metastasis, whole-brain radiotherapy, stereotactic radiosurgery, stereotactic radiotherapy

## Abstract

Background: Differences in radiation-induced lymphopenia and prognosis between methods of radiotherapy (RT) for brain metastases remain unclear. Methods: In this retrospective analysis of patients who underwent whole-brain radiotherapy (WBRT) or stereotactic radiosurgery/radiotherapy (SRS/SRT) for brain metastases, baseline total lymphocyte count (TLC) data were obtained within 2 weeks before RT initiation. Follow-up TLC data were evaluated at 0–2, 2–4, and 4–8 weeks after RT completion. Persistent lymphopenia was defined as <800/μL at any time point. Results: Overall, 138 RT courses in 128 patients were eligible (94 WBRT; 44 SRS/SRT). In the WBRT courses, the median baseline TLC was 1325/μL (IQR: 923–1799). Follow-up TLC decreased significantly to 946/μL (626–1316), 992/μL (675–1291), and 1075/μL (762–1435) (*p* < 0.001). SRS/SRT courses showed no significant TLC decrease. Multivariate analysis revealed female sex, prior RT, baseline TLC < 800/μL, and WBRT use were significantly associated with persistent lymphopenia. In the WBRT group, overall survival was significantly different between those with and without persistent lymphopenia (median, 2.6 and 6.1 months; *p* < 0.001). However, there was no significant difference in survival in the SRS/SRT group (*p* = 0.60). Conclusion: This study suggests SRS/SRT might be preferable for lymphocyte preservation in brain metastasis patients.

## 1. Introduction

Approximately 20–40% of patients with cancer develop brain metastases [1]. As the overall survival (OS) of patients with cancer increases with the development of treatments for malignant tumors and diagnostic techniques, such as MRI, for detecting small lesions, the number of patients with brain metastases is expected to increase. Metastatic brain tumors, depending on their location, can lead to paralysis and consciousness disorders, affecting survival and quality of life. Radiotherapy (RT) is crucial in the treatment of metastatic brain tumors, either alone or in combination with surgery or systemic therapy [2].

There are two major methods of RT for metastatic brain tumors: stereotactic radiosurgery (SRS) or stereotactic radiotherapy (SRT) and whole-brain radiotherapy (WBRT). SRS/SRT irradiates only visible tumors and provides a high degree of local control and preservation of normal organs. It is used for a small number of lesions, with up to 10 being irradiated [3]. However, when there are >10 lesions or cases of leptomeningeal metastasis, WBRT is performed. WBRT is typically conducted using conventional irradiation methods with two opposing beams. Although the WBRT method is simple and easy to perform for patients in poor health, it can cause adverse events, including dermatitis, alopecia, dry mouth, otitis media, and impaired cognitive function.

Total lymphocyte count (TLC) serves as a prognostic biomarker for patients with cancer. Higher peripheral TLC in breast cancer and recurrent esophageal cancer is associated with lower mortality [4,5,6,7]. Conversely, lymphopenia is associated with poor OS in patients with cancer [5]. RT causes lymphopenia because of the high radiosensitivity of lymphocytes. According to a systematic review and meta-analysis of patients with solid tumors treated with radiotherapy, radiation-induced lymphopenia is associated with OS [8]. Regarding WBRT for metastatic brain tumors from small-cell lung cancer, treatment-related lymphopenia is associated with prognosis [9]. However, the association between radiation-induced lymphopenia and OS in patients undergoing RT, including WBRT and SRS/SRT for metastatic brain tumors, remains unclear.

Consequently, this study aimed to investigate the temporal changes in TLC, the impact of radiation-induced lymphopenia on the survival of patients receiving RT for brain metastasis, and the differences in TLC between patients receiving WBRT and those undergoing SRS/SRT.

## 2. Materials and Methods

### 2.1. Patients

Patients aged 18 years or older who underwent WBRT or SRS/SRT for brain metastasis between January 2019 and December 2021 were retrospectively reviewed. For patients with hematologic malignancy as the primary disease, RT courses without baseline TLC data obtained within 2 weeks before the start of RT or obtained without any follow-up TLC data within 8 weeks after the completion of RT were excluded. Patients who underwent prophylactic cranial irradiation (PCI) during the same period were included in the exploratory analysis. The study protocol was approved by the Ethics Committee of Kindai University Hospital (R05-160) on December 26, 2023. The requirement for written informed consent was waived because of the retrospective design of the study.

### 2.2. Treatments

WBRT was selected for patients with more than 10 lesions, leptomeningeal metastases, or lesions larger than 3.0 cm. For WBRT, we prescribed 30 Gy in 10 fractions (Fr). Patients who appeared to have a better prognosis received a dose of 37.5 Gy in 15 Fr at the discretion of the staff radiation oncologist. If a brain lesion was single and <1.5 cm, we used SRS. For lesions with diameters between 1.5 cm and 3.0 cm, brainstem lesions, or lesions requiring reirradiation, SRT was used. SRT was also utilized in postoperative cases even if the surgical cavity was >3.0 cm. Until February 2021, we used the following dose regimen for SRS and SRT: 25 Gy in 1 Fr, 30 Gy in 3 Fr, or 36 Gy in 6 Fr to D80 (dose covering 80% of the target volume). In March 2021, the prescription doses were changed as follows: 20 Gy in 1 Fr, 24 Gy in 3 Fr, or 28.8 Gy in 6 Fr to D99.5.

### 2.3. Data Collection

The following patient characteristics, tumor characteristics, and radiotherapy details were retrospectively collected from medical records and used for analysis: age, sex, Karnofsky performance status (KPS), history of RT, systemic therapy before RT, primary tumor site, number of brain metastases, extracranial cancer, baseline and post-RT TLCs, methods of RT, concurrent extracranial RT, concurrent systemic therapy with RT, and steroid use during RT. Systemic therapy before RT was defined as having received any systemic therapy in the 60 days prior to RT. Follow-up TLC data for each course were evaluated at three time points: 0–2, 2–4, and 4–8 weeks after the completion of RT. If multiple TLC data points were available at each time point, the average value was used. Persistent lymphopenia was defined as having <800/μL at any time point.

### 2.4. Statistical Analysis

Follow-up TLC at each time point was compared with baseline TLC using the Mann–Whitney U test. *p*-values were adjusted using the Holm method for multiple comparisons of TLC. Univariate logistic regression analysis was used to identify factors associated with persistent lymphopenia, followed by multivariate logistic regression, including factors with *p* < 0.2 in the univariate analysis. OS was estimated as the time from the first course of brain RT during the study period to the date of death or the last visit using the Kaplan–Meier method, and the log-rank test was used to compare OS between the WBRT and SRS/SRT groups. Statistical significance was set at *p* < 0.05. Statistical analyses were performed using the R software (version 4.2.2).

## 3. Results

### 3.1. Patient Characteristics and Treatment Information

**Figure 1:**
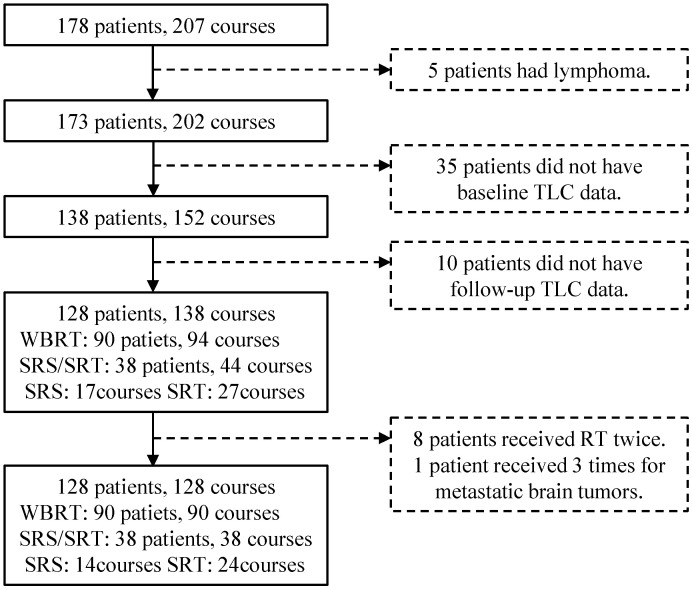
Flowchart of patient inclusion and exclusion. Abbreviations: TLC, total lymphocyte count; RT, radiotherapy; SRS/SRT, stereotactic radiosurgery or stereotactic radiotherapy; WBRT, whole-brain radiotherapy.

In total, 178 patients underwent RT for metastatic brain tumors, with 207 courses of treatment during the study period. Five courses were excluded, as the primary disease was lymphoma. Additionally, 64 courses in 45 patients were excluded because of the lack of TLC data within 2 weeks before the start of RT or 8 weeks after the end of RT. Finally, 138 courses in 128 patients were included in this study. Of the 138 courses, 94 were WBRT, and 44 were SRS/SRT (Figure 1).

During the first course of RT for brain metastases, 90 and 38 patients underwent WBRT and SRS/SRT, respectively. Patient characteristics are shown in Table 1. There were 48 patients (53%) who received WBRT at a prescribed dose of 30 Gy in 10 Fr, and 24 patients (27%) received WBRT and were prescribed 37.5 Gy in 15 Fr. Sixteen patients (42%) received SRS, and twenty-two (58%) received SRT.

### 3.2. Changes in TLC and Lymphopenia-Related Factors

**Figure 2:**
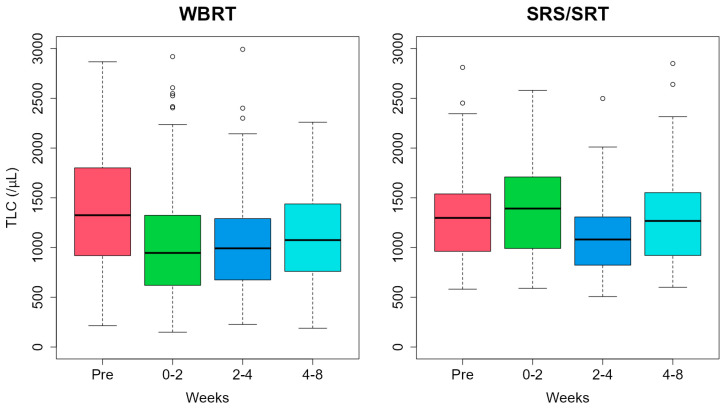
Temporal changes in total lymphocyte count (TLC). Abbreviations: Pre, baseline total lymphocyte count; SRS/SRT, stereotactic radiosurgery or stereotactic radiotherapy; WBRT, whole-brain radiotherapy.

The median (interquartile ranges [IQRs]) of the baseline TLC were 1325 (923–1799)/µL and 1298 (978–1524)/µL for WBRT and SRS/SRT, respectively. TLC values in WBRT were 946 (626–1316)/µL, 992 (675–1291)/µL, and 1075 (762–1435)/µL at 0–2 weeks, 2–4 weeks, and 4–8 weeks after brain RT, respectively (Figure 2). In the WBRT courses, significant decreases in TLC were observed at all time points (*p* < 0.001, *p* < 0.001, and *p* = 0.008, respectively). In the SRS/SRT courses, TLC values were 1392 (992–1705, *p* = 0.71)/µL, 1081 (823–1308, *p* = 0.18)/µL, and 1268 (921–1552, *p* = 0.79)/µL at 0–2 weeks, 2–4 weeks, and 4–8 weeks, respectively.

Univariate analysis of 128 patients showed that persistent lymphopenia was significantly associated with female sex, lower KPS scores, and prior history of RT and WBRT (Table 2). Multivariate analysis showed that female sex (odds ratio [OR], 3.72; 95% confidence interval [CI], 1.21–11.40; *p* = 0.022), prior history of RT (OR, 3.91; 95% CI, 1.27–12.10; *p*  = 0.018), baseline TLC < 800/μL (OR, 14.40; 95% CI, 3.57–58.20; *p* < 0.001), and the utilization of WBRT (OR, 6.25; 95% CI, 1.23–31.70; *p* = 0.027) were significantly associated with persistent lymphopenia.

### 3.3. Overall Survival

**Figure 3:**
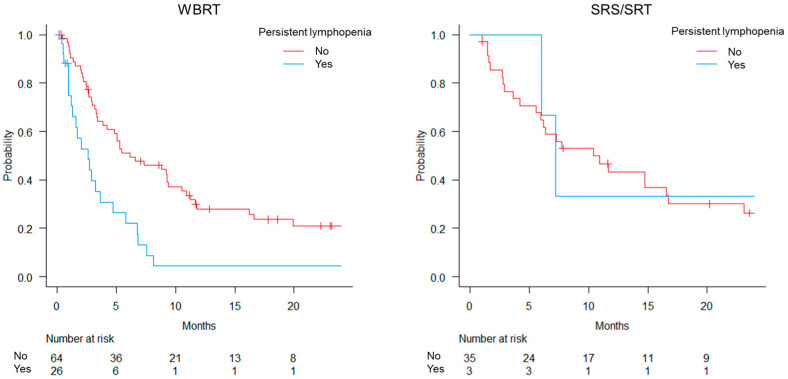
Overall survival for whole-brain radiotherapy (WBRT) and stereotactic radiosurgery or stereotactic radiotherapy (SRS/SRT).

The median potential follow-up according to the reverse Kaplan–Meier method was 23.1 and 31.1 months for the WBRT and SRS/SRT groups, respectively (Figure 3). Persistent lymphopenia was observed in 26 (29%) and 3 (8%) patients in the WBRT and SRS/SRT groups, respectively (Tables S1 and S2). In the WBRT group, the OS was significantly worse in patients with persistent lymphopenia than in those without, with median survival times of 2.6 and 6.1 months, respectively (*p* < 0.001). The median survival times in the SRS/SRT group were 7.2 months for those with persistent lymphopenia and 11.7 months for those without (*p* = 0.60).

### 3.4. PCI

In total, 21 patients received PCI, and 14 courses of PCI were included in the study (Figure S1 and Table S3). All patients were administered 25 Gy via 10 Fr. 

The median of the baseline TLC was 1216 (800–1776)/µL for PCI (Figure S2). The PCI courses showed no significant change in TLC at any time point with median IQRs of 1047 (752–1357, *p* = 1.00)/µL, 1031 (673–1974, *p* = 1.00)/µL, and 1170 (723–1624, *p* = 1.00)/µL at 0–2 weeks, 2–4 weeks, and 4–8 weeks, respectively.

## 4. Discussion

This study compared lymphopenia after different irradiation methods with respect to brain metastases. In the WBRT courses, significant decreases in TLC were observed after the completion of RT; however, no significant declines were observed in the SRS/SRT courses. Persistent lymphopenia after WBRT was associated with poor prognosis. Lymphopenia after RT was associated not only with WBRT but also with female sex, low KPS, a history of radiation therapy, and baseline TLC < 800/µL. To the best of our knowledge, this is the first study to compare WBRT and SRS/SRT for the treatment of brain metastases associated with RT-induced lymphopenia.

Some studies have evaluated brain-irradiation-induced lymphocytopenia [9,10,11,12]. WBRT for brain metastases from small-cell lung or breast cancer induces lymphopenia, which serves as a prognostic factor [9,12]. Byun et al. and Yovino et al. revealed that increased planning target volume (PTV) is associated with increased severe lymphopenia in patients receiving chemoradiation for glioblastoma [10,11]. The irradiation of the lymphoid organs, bone marrow, and circulating lymphocytes may induce lymphopenia. Approximately 2.9% of marrow exists in the skull, and the brain has few lymphatic vessels [13]. In contrast, the brain receives approximately 12% of the cardiac output [14]. Yovino et al. found that 99% of circulating lymphocytes received ≥0.5 Gy due to RT for malignant gliomas with 30 Fr of 2 Gy, resulting in a decrease in the number of lymphocytes [11]. Considering the findings of previous studies, SRS/SRT with a smaller PTV is preferable for WBRT to preserve lymphocytes. The results of the present study showed that patients in the PCI group did not show any significant differences in TLC at any time point, suggesting that lower prescribed doses may prevent lymphocyte depletion. 

In addition to WBRT, persistent lymphopenia after RT for brain metastasis was associated with lower baseline TLC, female sex, and prior history of RT. A lower baseline TLC has been identified as a risk factor for radiation-induced lymphopenia in several studies and may also be a prognostic factor after RT [15]. Doi et al. revealed that a baseline neutrophil-to-lymphocyte ratio < 5.0, which reflects higher TLC, is related to prolonged survival after WBRT for non-small-cell lung cancer [16]. Remarkably, several studies of RT for glioma have reported that female sex is associated with an increased risk of lymphopenia [17,18,19,20]. Based on this study and previous reports, the female sex seems to be strongly associated with lymphopenia, although the reason for this is unclear. The present study suggests that lymphopenia is associated with a history of RT, regardless of whether the head has been irradiated. Among the 28 patients with a history of RT in the WBRT group, 14 (50%) developed persistent lymphopenia. Previous irradiation continued the bone marrow suppression of lymphocyte production, which may make it difficult to maintain TLC.

Systemic therapy and steroids can affect TLC. However, neither concurrent systemic therapy nor steroid use during RT was found to be associated with persistent lymphopenia in this study. Several previous studies have shown similar results, finding that steroid administration is not associated with lymphopenia in RT for glioblastoma [19,20]. Furthermore, steroid use and concurrent chemotherapy administration are not associated with treatment-related lymphopenia in WBRT for small-cell lung cancer [9]. The various systemic therapy regimens and steroid doses in this study may have influenced their correlation with persistent lymphopenia after RT. Radiation-induced lymphopenia might mask the effect of steroids and systemic therapy on lymphocytes.

Recently, the number of malignancies for which immune checkpoint inhibitors (ICIs) are applicable has increased because ICIs can improve the prognosis of patients with cancer. A TLC of 1000/μL or higher before the administration of nivolumab for non-small-cell lung cancer patients tends to be associated with better progression-free survival and is significantly associated with a better OS [7]. In addition to lung cancer, metastatic triple-negative breast cancer, and recurrent esophageal cancer treated with ICIs, higher peripheral blood lymphocyte counts are associated with a better OS [5,6]. Schweiger et al. reported that RT for glioblastoma increased the expression of PD-L1 in tumor-associated macrophages and discussed the need to combine RT with ICIs [21]. Pike et al. demonstrated that patients treated with palliative extracranial RT had lymphopenia and that RT-induced lymphopenia decreased OS in patients receiving PD-1-directed ICI [22]. However, Benitez et al. reported that lymphopenia after SRS was associated with worse OS in patients treated with SRS and ICIs for brain metastases [23]. Considering the use of ICIs, SRS/SRT, which is less likely to cause lymphopenia, is more appropriate than WBRT.

Our study has some limitations. First, as this was a retrospective study, there was a potential source of selection bias. The choice of RT modality (WBRT or SRS/SRT) was based on the discretion of the treating physician. Additionally, the number of patients with baseline and post-RT TLC data in this study was limited. Moreover, some patients received several courses of chemotherapy, which may have affected the lymphopenia and OS data. Future studies are warranted to validate the results of this study in a prospective setting. 

## 5. Conclusions

Lymphopenia was observed after WBRT and persisted for >4 weeks after the completion of RT. Persistent lymphopenia after WBRT is associated with a poor prognosis. Female sex, history of radiation therapy, lower baseline TLC, and WBRT were associated with post-RT lymphopenia. The results of this retrospective study suggest that SRS/SRT is preferable for lymphocyte preservation in patients with brain metastases.

## Figures and Tables

**Table 1:** Patient characteristics.

Factor		Patients *n* = 128
Age, years (range)		70 (34–87)
Sex	Male	88
	Female	40
KPS	0–60	35
	70–80	37
	90–100	56
Primary tumor site	Lung (NSCLC or NOS)	55
	Lung (small cell)	33
	Breast	9
	Stomach	6
	Colon and rectum	4
	Others	21
Prior history of RT	None	83
	Cranial	8
	Extra-cranial	37
Prior RT courses	1 course	35
	2 courses	7
	3+ courses	3
Methods of RT	WBRT	90
	SRS/SRT	38
Systemic therapy before RT	No	53
	Yes	75
Concurrent extra-cranial RT	No	125
	Yes	3
Concurrent systemic therapy	No	114
	Yes	14
Steroid use during RT	No	57
	Yes	71

Abbreviations: KPS, Karnofsky performance status; NOS, not otherwise specified; NSCLC, non-small-cell lung cancer; RT, radiotherapy; SRS/SRT, stereotactic radiosurgery or stereotactic radiotherapy; WBRT, whole-brain radiotherapy.

**Table 2:** Univariate and multivariate analysis for persistent lymphopenia after RT.

				Univariate Analysis		Multivariate Analysis	
Characteristic		Yes	No	OR [95% CI]	*p*-Value	OR [95% CI]	*p*-Value
Age	<70 years	14	48	Reference	0.98		
	≥70 years	15	51	1.01 [0.44–2.31]			
Sex	Male	14	74	Reference	0.008 *	Reference	0.022 *
	Female	15	25	3.17 [1.34–7.48]		3.72 [1.21–11.40]	
KPS score	90–100	6	50	Reference	<0.001 *	Reference	0.23
	70–80	10	27	3.09 [1.01–9.41]		2.20 [0.56–8.56]	
	0–60	13	22	4.92 [1.66–14.60]		3.16 [0.83–12.00]	
History of RT	No	12	71	Reference	0.004 *	Reference	0.018 *
	Yes	17	28	3.59 [1.52–8.48]		3.91 [1.27–12.10]	
Number of brain metastasis	1	6	25	Reference	0.74		
	2–3	6	24	1.04 [0.29–3.68]			
	≥4	17	50	1.42 [0.50–4.04]			
Extracranial cancer	No	9	33	Reference	0.82		
	Yes	20	66	1.11 [0.46–2.71]			
Systemic therapy before RT	No	10	43	Reference			
	Yes	19	56	1.49 [0.63–3.52]			
TLC before RT	≥800/µL	15	94	Reference	<0.001 *	Reference	<0.001 *
	<800/µL	14	5	17.50 [5.51–55.80]		14.40 [3.57–58.20]	
Methods of RT	SRS/SRT	3	35	Reference	0.016 *	Reference	0.027 *
	WBRT	26	64	4.74 [1.34–16.80]		6.25 [1.23–31.70]	

Abbreviations: CI, confidence interval; KPS, Karnofsky performance status; OR, odds ratio; RT, radiotherapy; SRS/SRT, stereotactic radiosurgery or stereotactic radiotherapy; TLC, total lymphocyte count; WBRT, whole-brain radiotherapy. The asterisks (*) indicate statistical significance.

## Data Availability

The raw data supporting the conclusions of this article will be made available by the authors upon request.

## Supplementary Materials

The following supporting information can be downloaded at https://www.mdpi.com/article/10.3390/curroncol31080340/s1: Table S1: Patient characteristics for WBRT. Table S2: Patient characteristics for SRS/SRT. Table S3: Patient characteristics for PCI. Figure S1: Flowchart of patient inclusion and exclusion for prophylactic cranial irradiation. Figure S2: Temporal changes in TLC.
