# Supplementary material for: Radiation-Induced Lymphopenia and Its Impact on Survival in Patients with Brain Metastasis
Source: Curr Oncol. 2024 Aug 9;31(8):4559–67. doi: 10.3390/curroncol31080340 (PMC11353846; doi:10.3390/curroncol31080340)
Supplement: Supplementary file 1 [file curroncol-31-00340-s001.zip › curroncol-3140018-supplementary.pdf]

Supplementary Materials

Table S1. Patient characteristics of WBRT.

Factor	Group	Overall	Persistent lymphopenia	
		n=90	Yes (n=26)	No (n=64)
Age, years (range)		70 (34–87)	71 (36–87)	70 (34–85)
Sex	Male	59	12	47
	Female	31	14	17
KPS	0–60	29	13	16
	70–80	29	9	20
	90–100	32	4	28
Primary tumor site	Lung (NSCLC or NOS)	36	8	28
	Lung (Small cell)	27	4	23
	Breast	7	2	5
	Stomach	5	3	2
	Colon and rectum	2	1	1
	Others	13	8	5
Prior history of RT	None	62	12	50
	Cranial	5	2	3
	Extra-cranial	23	12	11
Prior RT courses	1 course	22	11	11
	2 courses	3	1	2
	3+ courses	3	2	1
Systemic therapy before RT	No	36	8	28
	Yes	54	18	36
Concurrent extra-cranial RT	No	88	25	63
	Yes	2	1	1
Concurrent systemic therapy	No	81	24	57
	Yes	9	2	7
Steroid use during RT	No	36	9	27
	Yes	54	17	37

Abbreviations: KPS, Karnofsky Performance Status; NSCLC, non-small cell lung cancer; NOS, not otherwise specified; RT, Radiotherapy; WBRT, whole brain radiotherapy

Table S2. Patient characteristics of SRS/SRT.

Factor	Group	Overall	Persistent lymphopenia	
		n=38	Yes (n=3)	No (n=35)
Age, years (range)		69 (42–83)	68 (63–74)	70 (42–83)
Sex	Male	29	2	27
	Female	9	1	8
KPS	0–60	6	0	6
	70–80	8	1	7
	90–100	24	2	22
Primary tumor site	Lung (NSCLC or NOS)	19	3	16
	Lung (Small cell)	6	0	6
	Breast	2	0	2
	Stomach	1	0	1
	Colon and rectum	2	0	2
	Others	8	0	8
Prior history of RT	None	21	0	21
	Cranial	3	0	3
	Extra-cranial	14	3	11
Prior RT courses	1 course	13	2	11
	2 courses	4	1	3
	3+ courses	0	0	0
Systemic therapy before RT	No	17	2	15
	Yes	21	1	20
Concurrent extra-cranial RT	No	37	3	34
	Yes	1	0	1
Concurrent systemic therapy	No	33	3	30
	Yes	5	0	5
Steroid use during RT	No	21	2	19
	Yes	17	1	16

Abbreviations: KPS, Karnofsky Performance Status; NSCLC, non-small cell lung cancer; NOS, not otherwise specified; RT, Radiotherapy; SRS/SRT, stereotactic radiosurgery or stereotactic radiotherapy

Table S3. Patient characteristics of PCI.

Factor	Group	PCI n=14
Age, years (range)		71.5 (56–84)
Sex	Male	12
	Female	2
KPS	0–60	0
	70–80	0
	90–100	14
Primary tumor site	Lung (Small cell)	14
Prior history of RT	None	3
	Cranial	0
	Extra-cranial	11
Prior RT courses	1 course	11
	2 courses	0
	3+ courses	0

Abbreviations: KPS, Karnofsky Performance Status; PCI, prophylactic cranial irradiation; RT, radiotherapy.

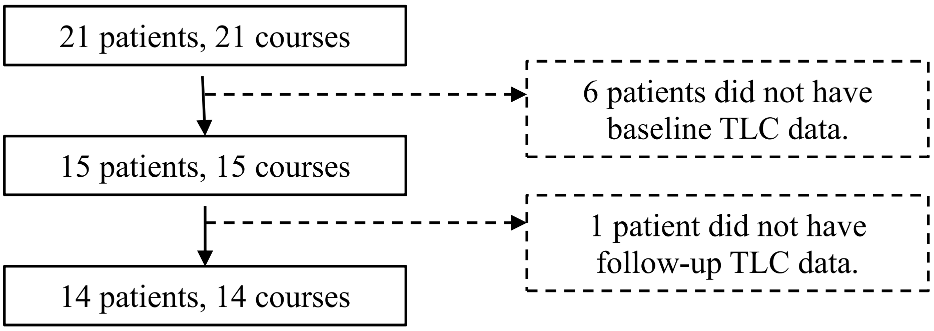

Figure S1. Flowchart of patient inclusion and exclusion for prophylactic cranial irradiation. Abbreviation: TLC, total lymphocyte count.

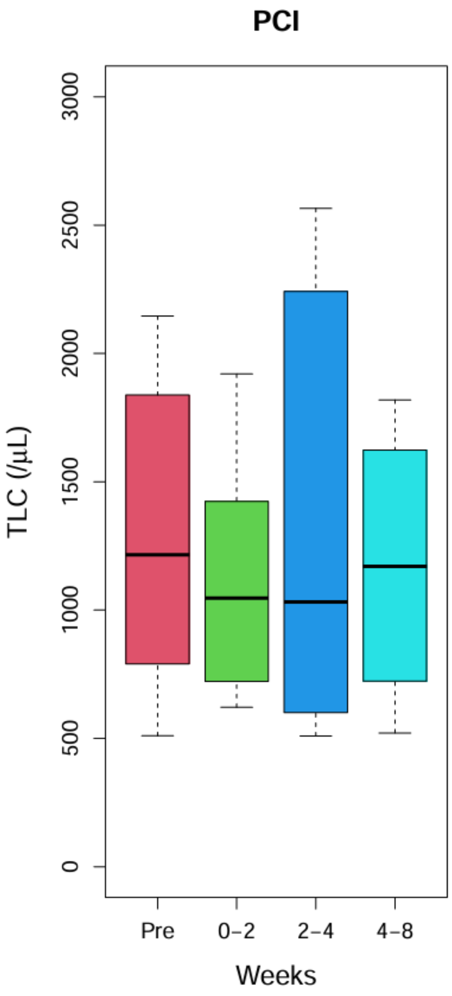

Figure S2. Temporal changes in TLC. Abbreviations: Pre, baseline total lymphocyte count; PCI, prophylactic cranial irradiation; TLC, total lymphocyte count.
